# Wavelet Scattering and Neural Networks for Railhead Defect Identification

**DOI:** 10.3390/ma14081957

**Published:** 2021-04-14

**Authors:** Yang Jin

**Affiliations:** Department of Structural Engineering, Delft University of Technology, Postbus 5, 2600 AA Delft, The Netherlands; J.Jin-3@tudelft.nl; Tel.: +31-06-2004-8305

**Keywords:** wavelet scattering networks, neural networks, railhead defect identification, machine learning

## Abstract

Accurate and automatic railhead inspection is crucial for the operational safety of railway systems. Deep learning on visual images is effective in the automatic detection of railhead defects, but either intensive data requirements or ignoring defect sizes reduce its applicability. This paper developed a machine learning framework based on wavelet scattering networks (WSNs) and neural networks (NNs) for identifying railhead defects. WSNs are functionally equivalent to deep convolutional neural networks while containing no parameters, thus suitable for non-intensive datasets. NNs can restore location and size information. The publicly available rail surface discrete defects (RSDD) datasets were analyzed, including 67 Type-I railhead images acquired from express tracks and 128 Type-II images captured from ordinary/heavy haul tracks. The ultimate validation accuracy reached 99.80% and 99.44%, respectively. WSNs can extract implicit signal features, and the support vector machine classifier can improve the learning accuracy of NNs by over 6%. Three criteria, namely the precision, recall, and F-measure, were calculated for comparison with the literature. At the pixel level, the developed approach achieved three criteria of around 90%, outperforming former methods. At the defect level, the recall rates reached 100%, indicating all labeled defects were identified. The precision rates were around 75%, affected by the insignificant misidentified speckles (smaller than 20 pixels). Nonetheless, the developed learning framework was effective in identifying railhead defects.

## 1. Introduction

With the continuous increase in the loading capacity and operational velocity of railway systems, frequent high-stress contacts exist between the train wheels and the tracks, which aggravate the deterioration and produce railhead defects [1,2]. Although material [3] and construction [4] technologies evolved to improve rail durability, surface damages by continuous fatigue, wear, and harsh environments are unavoidable [5,6,7]. If not addressed, the initial damages will develop into severe transverse defects, including squats, head checking, and gauge corner collapse [8], which have resulted in numerous derailment incidents [9]. Therefore, a non-destructive technology to discover the surface defects is significant for the security of railway systems. Contact measurement instruments, including acceleration sensors and ultrasound techniques, have limitations as mass loading affects the modal analysis [10]. Although interpreting signals from acceleration sensors can identify the existence of track defects, the further extraction of accurate size information is difficult (e.g., [11,12]). The detection error of ultrasound techniques is large with certain crack angles or defect sizes smaller than 5% of the railhead area [13]. In contrast, some non-contact technologies like cameras can continuously capture the pictures that record the locations and sizes of the defects [14]. The significant issue is that complicated background noises pollute the defect images. Diverse hand-crafted processing approaches (e.g., [15,16]) have been developed to extract defect features, but are either sensitive to noises or dependent on expert experience. A coarse-to-fine model was established by Yu et al. [17] for defect detection, but with several significant parameters determined by experience. Therefore, automatic and applicable signal interpretation approaches have been subjected to extensive investigations.

Machine learning provides automatic computational learning approaches that require no human intervention or assistance [18]. Without being explicitly programmed, the learning algorithms acquire the joint distribution of input and output variables based on the sampled dataset. Recent research has successfully identified and predicted structural defects by feeding mechanical information into artificial neural networks [19,20]. By learning on images, deep learning approaches can properly classify pipelines [21] and identify oil spills [22]. For railhead defect analysis, several learning frameworks have been investigated, e.g., a machine vision approach for identifying railhead defects [23], deep convolutional neural networks (CNNs) for classifying rail surface defects [24], and deep CNNs for segmentation of defect images [25]. Although automatic classification provides the information of defect patterns and image segmentation identifies approximate defect locations, the defect sizes have not been calculated. The defect sizes affect the remaining fatigue life of steel materials [26], thereby the related calculation becomes significant. Zhuang et al. [27] proposed a double-layer framework to extract defect boundaries, but the railhead edges were sometimes misidentified as the defects. Another issue restricting the deep learning approaches is the dataset requirement, as the data should be sufficient to train the numerous parameters. Convolutional networks failed to capture the defect features due to the limited dataset [27], while manually labeling over 20,000 samples was time consuming [24]. Yuan et al. [28] fed over 180,000 samples to train the deep CNN, but only obtained a classification accuracy of 87%. Therefore, a machine learning approach, which is functionally equivalent to a deep CNN, should be investigated for identifying both defect locations and sizes.

Wavelet scattering networks (WSNs) are convolutional networks that are structurally similar to CNNs. They were originally proposed by Mallat [29] to realize translation and rotation invariance. In the WSN framework, each layer contains linear and nonlinear operators corresponding to convolution and activation operators of CNNs, respectively. Therefore, WSNs become functionally equivalent to deep CNNs. Specifically, the linear operator is predefined by the wavelet groups, performing as band-pass filters to extract physical features. Since all operators have been predefined, the WSN contains no parameters, and thus, the dataset requirement decreases. In [30], only 40 radargrams were required for training and validation to achieve a learning accuracy over 95%. The learning frameworks based on WSNs also outperformed CNNs in multiple applications, achieving a learning accuracy up to 99.7% (e.g., [31,32]). However, the nonlinear operator eliminates the location information, and other strategies should complement the learning framework for identifying defect locations and sizes. Neural networks (NNs) can reveal the internal relationships between the variables and the hidden information of the signals [33]. In deep learning, NNs behave as fully connected layers for extracting representative characteristics [34] or decoders for restoring the original information [35]. Therefore, they have the potential to recover the location information. To our best knowledge, no research has investigated WSNs assisted by NNs for detecting the defects especially on railheads, which is the concern of our research.

In this paper, a machine learning framework based on WSNs and NNs is developed for identifying both the locations and sizes of railhead defects. The railhead damages concerned in this research are produced by rolling contact fatigue, including squats and other related defects. The topological relationship determines the learning effectiveness, and thereby, different frameworks are compared to evaluate our learning components. The remainder of this paper is organized as follows: Section 2 describes the adopted data and the developed learning framework; Section 3 presents the results and compares with other approaches; Section 4 discusses and concludes this paper.

## 2. Materials and Methods

### 2.1. Data Description

**Figure 1:**
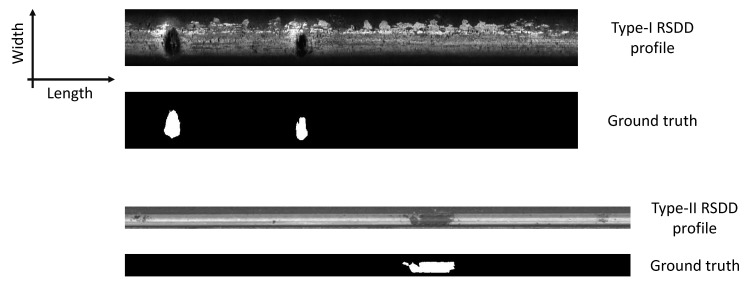
Representative railhead profiles and corresponding ground truth images (data value, black: 0; white: 255). RSDD, rail surface discrete defects dataset.

The publicly available data resource namely the rail surface discrete defects (RSDD) dataset [36], which has been extensively applied in evaluating different approaches for railhead defect detection (e.g., [17,37,38]), constituted the data foundation of this research. It comprises two types of track surface images: the Type-I RSDD dataset contained 67 challenging images acquired from express tracks, and the Type-II RSDD dataset contained 128 challenging images acquired from ordinary/heavy haul tracks. Each surface profile contained one or more defects that were difficult to identify owing to the noisy backgrounds. These defects included squats at different levels and other related damages produced by rolling contact fatigue. Other important defects, like rail corrugation and railhead wear, were not concerned in the RSDD dataset. Crossings and turnouts were also excluded. Experienced experts have exported the actual defect locations into the corresponding “ground truth” images [36], which can work as accurate outputs to supervise our machine learning networks. The ground truth images contain pixel values of 0 at damage locations and 255 at other locations, different from the actual railhead pictures. Therefore, the labeled defect information only contains the surface sizes (lengths along the track direction and widths) except the depths and other parameters. As shown in Figure 1, the length and width coordinates are both on the top surface of the railhead, and we were concerned with the 2-dimensional analysis on the railhead top surface. The length coordinate represents the distance (in pixel levels) along the running direction of railways, and the width coordinate represents the distance perpendicular to the length. These coordinates are used in the rest of this paper. To regularize the learning inputs, images in the individual dataset should be resized to the same pixels. The images in the Type-II RSDD dataset already had the same pixels of 1250×55, while those in the Type-I RSDD dataset were resized to the pixels of 1000×160. The resolution (1 × 1 pixel) of Type-II images was 1 mm × 1 mm [15], and thereby, the real size was 1250 mm × 55 mm. The resolution and real size of Type-I images were not available in the literature. Two representative railhead profiles and corresponding ground truth images are presented in Figure 1.

### 2.2. Wavelet Scattering Networks

Three significant components constituted our learning framework. The first one was the wavelet scattering network (WSN). WSNs are convolutional networks developed based on the wavelet transform, which is the first procedure of WSNs to decompose the signals in multiple directions [29]. Different from the Fourier transform acquiring signal frequencies, the wavelet transform localizes the signals in both the frequency and time domains. The wavelets are predefined local waveforms for convolution calculation with the signals, behaving as band-pass filters to decompose signals within certain bandwidths. Therefore, the wavelets are functionally equivalent to the convolution kernels of CNNs. This also determines that the WSN contains no parameters. The wavelet decomposition is complete and reversible to extract all signal characteristics, different from CNNs that adjust the kernels to extract targeted features. A wavelet group can be acquired by dilatation and rotation of the mother wavelet.

(1) $$\psi_{2^{-j}r}\left(t\right)=2^{-dj}\psi \left(2^{-j}r^{-1}t\right)$$

where $\psi \in L^{2}\left(R^{d}\right)$ is the mother wavelet, $2^{j}$ represents the dilation rate, and *r* is the rotation coefficient. By convolution calculation, the wavelets $\psi_{2^{-j}r}\left(t\right)$ work as band-pass filters to acquire the signal components.

(2) $$W_{\lambda }x=x⊗\psi_{\lambda }=\int x\left(\tau \right)\psi_{\lambda }\left(t-\tau \right)d\tau$$

where we simplify the notation as $\lambda =2^{-j}r$, $W_{\lambda }$ represents the wavelet transform operator, and ⊗ represents the convolution calculation.

However, the wavelet transform is translation covariant, while objects should be recognizable regardless of the location and orientation. Mallat [29] demonstrated that $L^{1}\left(R^{2}\right)$ norms can produce translation-invariant coefficients and thereby introduced a nonlinearity with modulus operators, which are functionally equivalent to the activation functions of CNNs. The iteration on the wavelet transform and modulus operators creates the scattering propagator of WSNs.

(3) $$U_{\lambda }x=|W_{\lambda }x|=|x⊗\psi_{\lambda }|$$

(4) $$U\left[p\right]=U_{\lambda_{m}}\ldots U_{\lambda_{2}}U_{\lambda_{1}}$$

(5) $$U\left[p\right]x=|||x⊗\psi_{\lambda_{1}}|⊗\psi_{\lambda_{2}}|\ldots ⊗\psi_{\lambda_{m}}|$$

where $U_{\lambda_{i}}$(i=1,2,⋯,m) is the comprehensive operator combining the wavelet transform and modulus and U[p] represents the scattering propagator.

**Figure 2:**
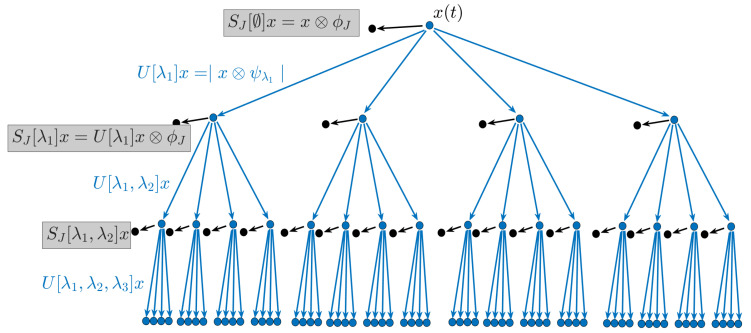
An example of WSNs. The first layer outputs $S_{J}\left[\emptyset \right]x$, where *∅* represents an empty set and operates $U_{\lambda_{1}}$ on the original signal *x*. Then, the second layer outputs $S_{J}\left[\lambda_{1}\right]x$ and operates $U_{\lambda_{2}}$ on the each previous result $U[\lambda_{1}]x$. The scattering procedure propagates iteratively to obtain all convolution results.

Till now, the wavelet scattering was not complete as the decomposition discarded signal components with the frequency $2^{-j}<2^{-J}$ ($2^{J}$ is the predefined transform scale, and the wavelet transform is only conducted with $2^{j}\geq 2^{J}$). Bruna and Mallat [32] considered keeping the spatial variability at scales larger than $2^{J}$, hence calculating the low-frequency components by convolution with a scaled spatial window.

(6) $$\phi_{J}\left(t\right)=2^{-dJ}\phi \left(2^{-J}t\right)$$

(7) $$S_{J}\left[p\right]x=|||x⊗\psi_{\lambda_{1}}|⊗\psi_{\lambda_{2}}|\ldots ⊗\psi_{\lambda_{m}}|⊗\phi_{J}$$

where ϕ is the original scaling function, which can be converted to the scaling function $\phi_{J}$ at the scale $2^{J}$, and $S_{J}\left[p\right]$ is the propagator to extend the frequency scale. By continuously calculating U[p]x and $S_{J}\left[p\right]x$, according to Equations (5) and (7), the WSNs can be constructed as shown in Figure 2.

The wavelet group and scaling functions are significant for constructing WSNs, and in this paper, Morlet wavelets and Gaussian windows (expressed in Equations (8) and (9), respectively) were adopted as the predefined convolution kernels.

(8) $$\psi \left(t\right)=K_{\sigma_{t}}e^{-\frac{t^{2}}{2\sigma_{t}^{2}}}e^{2\pi ift}$$

(9) $$w\left(t\right)=e^{-\frac{t^{2}}{2\sigma^{2}}}$$

where $K_{\sigma_{t}}$ is the normalization constant with the wavelet duration $\sigma_{t}$, *i* represents the imaginary unit, *f* is the normalized frequency, and σ is the standard deviation of a Gaussian random variable.

### 2.3. Neural Networks

**Figure 3:**
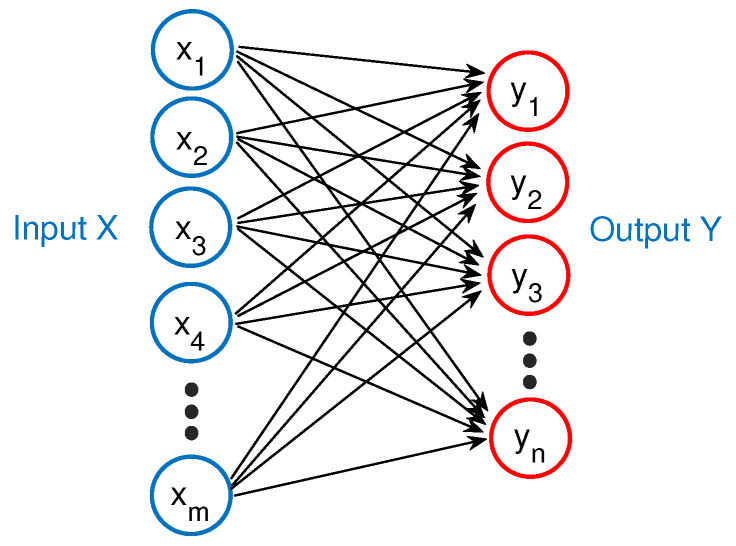
The two-layer NN structure utilized in this paper.

The second significant component is the neural network (NN). NNs constitute a self-adaptive approximator for nonlinear functions to learn the statistical relationships among variables [33]. Different from logistic regression, NNs utilize a multi-layer structure imitating biological neural networks to construct the nonlinear statistical model. The numbers of neurons and hidden layers determine the model complexity, thereby NNs becoming suitable for both simple and complicated issues. Since effective learning on non-intensive datasets was pursued in this paper, NNs without hidden layers (Figure 3) are adopted to minimize the parameters. The input layer consists of numerous neurons $X=\{x_{1},x_{2},\ldots ,x_{m}\}$ representing the input characteristics, and this model represents a nonlinear function $f\left(\cdot \right):R^{m}\to R^{n}$ to acquire the output $Y=\{y_{1},y_{2},\ldots ,y_{n}\}$. The specific output dimensions are 1250 and 1000 for the Type-I and Type-II datasets, respectively. Each neuron in the output layer is transformed from the input values with two steps, a weighted linear summation expressed in Equation (10) and a non-linear activation function expressed in Equation (11) (specified in this research).

(10) $$a_{k}=\omega_{1,k}x_{1}+\omega_{2,k}x_{2}+\ldots +\omega_{m,k}x_{m}+b_{k}$$

(11) $$y_{k}=\left\{\begin{array}{c} 0,\;a_{k}\leq 20\\ 255,\;a_{k}>20 \end{array}\right.,\;k=1,2,\ldots ,n$$

where $\omega_{j,k}$ is the weight parameter and $b_{k}$ is the bias parameter. The core of training NNs is to adjust these parameters for better data fitting.

The loss function is an indicator to evaluate the training model, as the loss becomes larger with worse data fitting. It calculates the function difference between the output *Y* and the actual value $Y^{\prime }$. The choice of loss functions can not only determine the model convergence, but also change the learning speed. In this paper, a modified “cross entropy” function is adopted as expressed in Equation (12).

(12) $$L=-\sum_{k=1}^{n}\left(\left(y_{k}+1\right)ln\left(y_{k}^{\prime }+1\right)+\left(256-y_{k}\right)ln\left(256-y_{k}^{\prime }\right)\right)$$

where $y_{k}^{\prime }$ is the true value corresponding to $y_{k}$. After calculating the losses, the loss function should provide the feedback for parameter adaption. The “stochastic gradient descent” approach is intended for updating the parameters according to the gradient of loss values.

(13) $$W\leftarrow W-\eta \frac{\partial L}{\partial W}$$

where $W=\{\omega_{l,k},b_{k},\;l=1,2,\ldots ,m,\;k=1,2,\ldots ,n\}$ represents the parameter set and η is the transmission speed.

### 2.4. Support Vector Machine

The third significant component is the support vector machine (SVM). SVMs are supervised learning models utilizing associated optimization algorithms for classification or regression analysis [39,40]. The SVM classifier is effective in high-dimensional applications even when the number of dimensions is greater than that of samples. This classifier was originally considered to solve linear problems, where a hyperplane for dividing data points into multiple categories was achieved at point-plane distance maximization. For efficiently performing nonlinear classification, kernel functions were considered to implicitly map the inputs into high-dimensional feature spaces. Therefore, the nonlinear solving algorithms were developed similar to linear classification. An appropriate kernel function is significant for improving effectiveness, and we utilized the most common one, namely the radial basis function, in this paper.

In binary classification, providing the input data pairs $T=\{\left(x_{1},y_{1}\right),\left(x_{2},y_{2}\right),\ldots ,\left(x_{N},y_{N}\right)\}$ (where $x_{k}\in R^{n}$, $y_{k}\in \left\{-1,1\right\}$, k=1,2,…,N), SVMs learn the classification criteria by solving the dual optimization problem [41].

(14) $$\begin{array}{cc} \; & \underset{\gamma }{min}\;\frac{1}{2}\gamma^{T}Q\gamma -O^{T}\gamma \\ \; & s.t.\;\;\;y^{T}\gamma =0\\ \; & 0\leq \gamma_{k}\leq C,\;k=1,2,\ldots ,N \end{array}$$

where γ is the dual coefficient vector upper-bounded by *C*, *O* is the vector of all ones, *Q* is a positive semidefinite matrix with $Q_{l,k}=y_{l}y_{k}K\left(x_{l},x_{k}\right)$, and $K(x_{l},x_{k})$ is the kernel function. The optimal solution $\gamma^{☆}=\left(\gamma_{1}^{☆},\gamma_{2}^{☆},\ldots ,\gamma_{N}^{☆}\right)$ is available after the learning procedure, which determines the classification hyperplane and the output decision function.

(15) $$g\left(x\right)=sgn(\sum_{k=1}^{N}\gamma_{k}^{☆}y_{k}K\left(x_{k},x\right)+b^{☆})$$

(16) $$b^{☆}=y_{l}-\sum_{k=1}^{N}\gamma_{k}^{☆}y_{k}K\left(x_{k},x_{l}\right)$$

where sgn represents the sign function $sgn\left(x\right)=\frac{x}{|x|}$ and $b^{☆}$ is the constant parameter of the hyperplane.

### 2.5. Learning Framework

**Figure 4:**
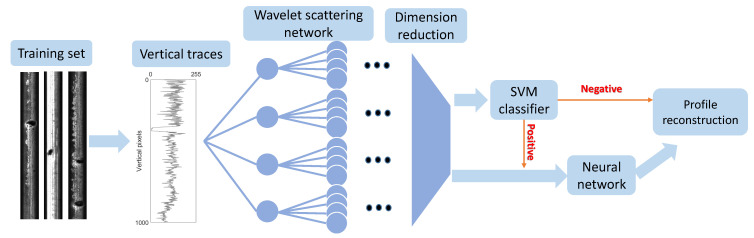
The machine learning framework for railhead defect identification.

A specific machine learning framework (Figure 4) was designed in this paper to identify the locations and sizes of railhead defects, different from the general learning architectures in the literature that overlooked the size information. Firstly, the 2D input training dataset was decomposed into 1D vertical traces (along the running direction), which can preserve horizontal labels (the trace location along the rail width) and extend the input dataset sufficiently for convincing learning. These vertical traces are then fed into the predefined WSNs to extract signal characteristics. The SVM classifier follows closely, identifying the existence of railhead defects on the vertical traces. Therefore, the vertical traces are divided into two categories, “positive” (existing defects) and “negative” (non-existing defects). As the negative traces contain no defect features while occupying a considerable amount, they will affect the learning model adversely, e.g., overlooking tiny defects. Under this consideration, the SVM classifier is significant in conveying positive traces to the NNs for further location and size identification. NNs were adopted to restore the ground truth traces from the positive traces, and the ground truth of negative traces equaled 0. Finally, the processed profiles containing location and size information were reconstructed by orderly merging of the output traces.

The input data pairs of the learning framework are ($x_{i},y_{i},z_{i}$), i=1,2,…, where $x_{i}$ is the vertical trace from actual images, $y_{i}$ is the corresponding trace from ground truth images, $z_{i}=0$ for negative traces, and $z_{i}=1$ for positive traces. The output of the SVM classifier is $z_{i}^{\prime }$ (the training result of $z_{i}$). The output of NNs is $\left\{y_{i}^{\prime }|z_{i}^{\prime }=1\right\}$ (the training result of $y_{i}$), and $\left\{y_{i}^{\prime }=0|z_{i}^{\prime }=0\right\}$ is achieved for profile reconstruction. For the Type-I dataset, the dimensions of $x_{i}$, $y_{i}$, and $y_{i}^{\prime }$ were 1000 × 1. For the Type-II dataset, the dimensions of $x_{i}$, $y_{i}$, and $y_{i}^{\prime }$ were 1250 × 1. Algorithm 1 provides the pseudocodes of the learning framework.


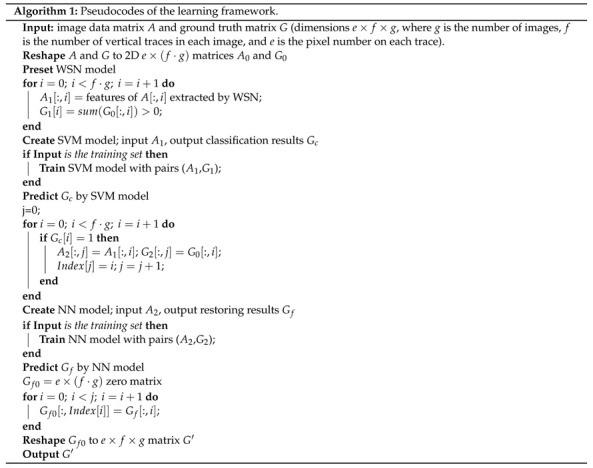


## 3. Results

### 3.1. Type-I RSDD Dataset

Firstly, the Type-I RSDD dataset containing 67 images captured from express tracks was analyzed to evaluate the developed learning framework. This dataset was randomly divided into 54 images for the training set and 13 images for the validation set, with each image resized to 1000 × 160 pixels. Therefore, we generated 67×160 signal traces with lengths (along the running direction of tracks) of 1000, which were sufficient to achieve reasonable results. Since photographing lights and railhead roughness influenced the image quality, it was significant that the training and validation sets contained both bright and dark images. Corresponding ground truth images were pre-processed by the same procedure to supervise our machine learning model. Three criteria were used to evaluate the learning results. The accuracy is the proportion of the same pixel values between reconstructed and ground truth images. The false positives (FPs) represent the pixels of zero miscalculated as 255. The false negatives (FNs) represent the pixels of 255 miscalculated as zero.

Classification is the crucial procedure to prepare positive traces for NNs, with the results shown in Table 1. Learning structures with and without WSNs were compared to evaluate the ability of feature extraction. Although the training accuracy rates of both structures reached 99.95%, the “WSN+SVM” model remarkably outperformed the independent SVM, improving validation accuracy up to 97.20%. The false rate of the WSN+SVM model was 2.80%, which means an average of 4.5 signal traces of each image were misclassified, and the effect of these insignificant errors is discussed in the profile reconstruction results. On the contrary, the validation accuracy of the independent SVM was 72.43%, over 27% less than its training accuracy, which means that the training set was excessively over-fitted. This unpromising result arose from the implicit signal features and complicated nonlinear relationships in the original training inputs. WSNs have presented a powerful ability to reveal the hidden signal characteristics, thereby achieving promising classification results. The WSNs cost 309.36 s of CPU time on a 1.90 GHz Intel i7-8650U CPU. The classification accuracy determined the effectiveness of NNs and the accuracy of profile reconstruction, and therefore, the independent SVM was not considered in further procedures.

Utilizing the prepared positive traces from the SVM classifier, NNs were trained to restore the ground truth traces. The inputs of NNs were feature-extracted and dimension-reduced signal series from WSNs, then sieved through the SVM. For comparison, another learning structure with NNs immediately following WSNs (no SVM for sieving positive traces) was considered to evaluate the effect of the SVM classifier. The learning accuracy is presented in Table 2. The SVM+NN model outperformed the NN model, improving the training accuracy up to 99.84% and the validation accuracy up to 99.47%. The small errors would affect some tiny defects. Although the model without the SVM achieved an accuracy of around 92%, the relatively grave errors would affect the identification of large defects. The false negative rate reached 6.09%, which means that numerous defects were overlooked by the NN model, since no classifier discarded the weighty, but feature-free negative traces. The SVM+NN model performed promisingly in restoring the ground truth traces, and the SVM classifier could increase the learning accuracy by 7.31%. The NN component cost 1781.1 s of CPU time. The learning accuracy determined the results of defect identification, and therefore, the NN model was not considered further to reconstruct the profiles.

The last procedure concerned reconstructing the inspection profiles and identifying the defects. Table 3 presents the learning accuracy by comparing the reconstructed profiles with the ground truth images. The validation accuracy remarkably reached 99.80%, indicating the effectiveness of our learning procedures. Although the WSN+SVM procedure had a 2.8% classification error, each misclassified trace affected less than 10 pixels, and thereby the effect on the whole framework was less than 2.8%×10/1000=0.028%. Although the SVM+NN procedure had a 0.53% error, the positive traces only accounted for around 30% of all traces, and thereby, the effect on the whole framework was around 0.53%×30%=0.159%. Therefore, the ultimate learning accuracy was higher than that of the partial procedures.

**Figure 5:**
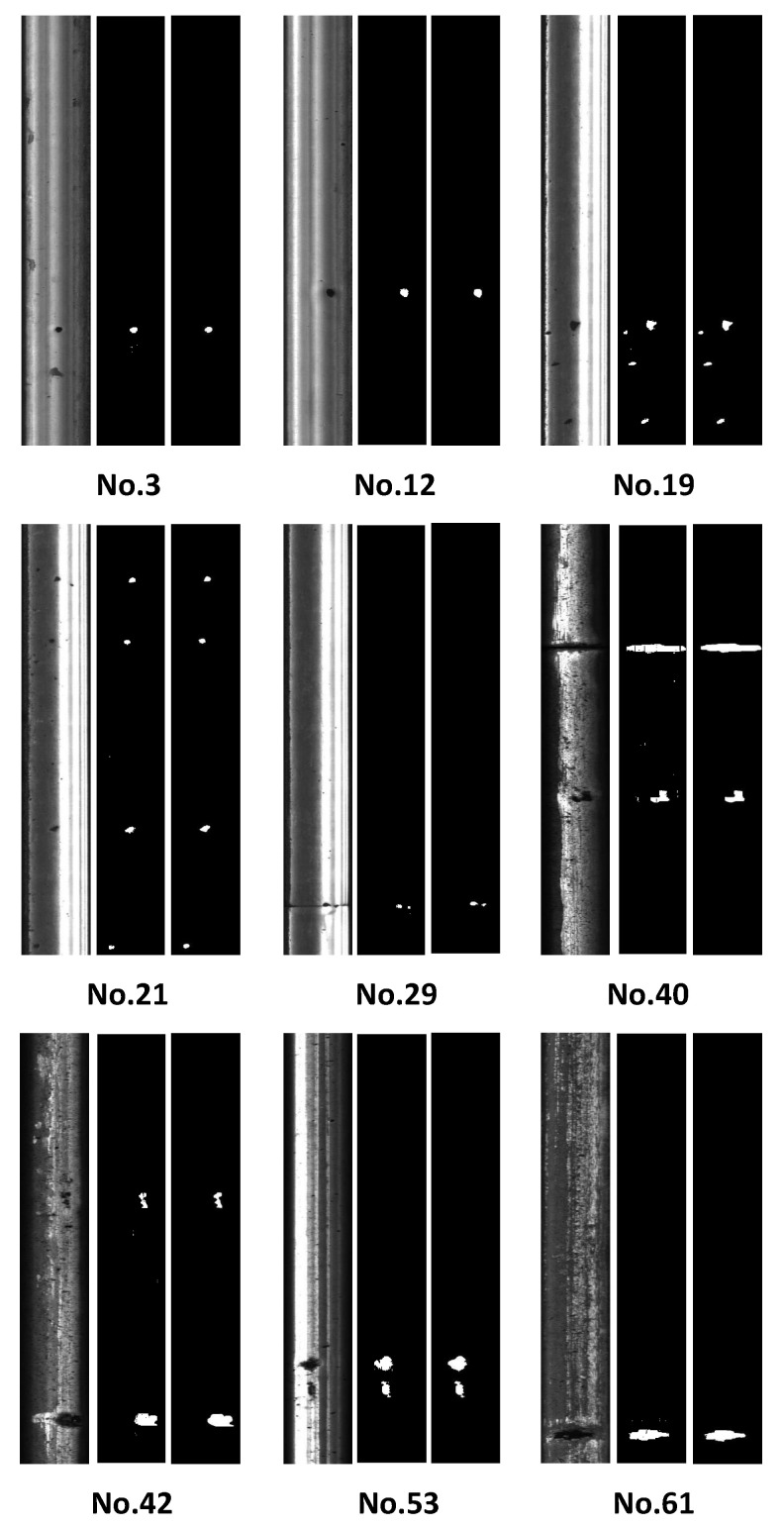
Example rails in the validation set, with label Nos. 3, 12, 19, 21, 29, 40, 42, 53, and 61 in the Type-I RSDD dataset. The sequence of each rail images is the original image, the learning result, and the ground truth.

Figure 5 illustrates the learning results of nine example rails in the validation set. The rail labels (Nos. 3, 12, 19, 21, 29, 40, 42, 53) are marked in the RSDD dataset. There were three kinds of original images with normal quality (Nos. 3 and 12), strong contrast (Nos. 19, 21, 29, and 53), and noisy background (Nos. 40, 42, and 61), respectively. After processing normal quality images, the reconstructed profiles corresponded well with the ground truth images despite the tiny shape differences of the defects. The identified defect sizes of the No. 3 and No. 12 rails were 195 and 298 pixels, respectively, while the actual sizes were 187 and 304 pixels, with size errors less than 4.3%. Central location coordinates (u,v) at the pixel level were used to evaluate the location accuracy (*u* is along the rail width, and *v* is along the running direction). The identified locations of the No. 3 and No. 12 rails were (87, 731.5) and (109, 646.5), respectively, while the actual locations were (86.5, 731.5) and (109, 646.5), with errors less than one pixel. Similar promising results were achieved in processing the strong-contrast images. Although some tiny defects were nearly invisible in the original images of the No. 19, 21, and 29 rails, the developed approach correctly identified the defect locations, corresponding well with the ground truth images. For example, the identified locations of four defects on the No. 21 rail were (83.5, 127.5), (72.5, 272.5), (79, 707.5), and (35.5, 980.5), while the actual locations were (84, 127.5), (72, 272), (78.5, 707.5), and (35.5, 980.5), with errors less than one pixel. The integral sizes of identified defects in the No. 19, 21, 29, and 53 rails were 667, 577, 148, and 1405 pixels, with errors of 1.3%, 3.6%, 7.5%, and 2.6%. The identification error increased when the defects were small. While processing original images with a noisy background, the reconstructed profiles also presented promising correspondence with the ground truth images. For example, the identified locations of defects on the No. 53 rail were (61, 769) and (65, 829.5), while the actual locations were (62, 770) and (66.5, 830), with errors less than two pixels. The integral sizes of identified defects in the No. 40, 42, and 61 rails were 2729, 1618, and 1538 pixels, with errors of 2.3%, 6.5%, and 1.0%. These promising results of different-quality images indicated that the developed learning framework was effective in identifying the sizes and locations of the railhead defects.

### 3.2. Type-II RSDD Dataset

Secondly, the Type-II RSDD dataset containing 128 images (1250 × 55 pixels) acquired from ordinary/heavy haul tracks was considered to evaluate the developed approach. This dataset was randomly divided into 102 images for the training set and 26 images for the validation set. The original images were reproduced to 128×55 signal traces with lengths (along the running direction of tracks) of 1250, which were sufficient to achieve reasonable results. Photographing lights influence the image quality, and the training and validation sets should contain both bright and dark images. Corresponding ground truth images were similarly pre-processed to supervise our machine learning model.

The starting procedure was classification to prepare positive traces for NNs, with the results shown in Table 4. The learning structures with and without WSNs, respectively, were compared to evaluate the ability of feature extraction. Although both learning models obtained a training accuracy over 99.5%, the WSN+SVM model remarkably outperformed the independent SVM, improving the validation accuracy to 94.74%. The false rate of the WSN+SVM model was 5.26%, which means an average of 2.9 signal traces of each image were misclassified, and the effect is discussed in the profile reconstruction results. The WSNs cost 319.44 s of CPU time. The learning accuracy of the Type-II dataset was smaller than that of the Type-I dataset, which may arise from the different image quality and resolution. Nonetheless, the promising classification results indicated that WSNs can effectively reveal the hidden signal characteristics. In contrast, the independent SVM was excessively over-fitted as the validation accuracy was 68.96%, over 30% less than the training accuracy. The classification accuracy determines the effectiveness of further procedures, and therefore, the independent SVM cannot be considered.

NNs were trained to restore the ground truth traces utilizing the positive traces sieved by the SVM. Another learning structure with NNs immediately following WSNs (no SVM for sieving positive traces) was compared with the learning accuracy presented in Table 5. The SVM+NN model outperformed the NN model, improving the training accuracy up to 99.77% and the validation accuracy up to 98.81%. The NNs cost 1596.3 s of CPU time. Affected by the weighty, but feature-free negative traces, the NN model overlooked numerous defects as the false negative rate reached 7.02%. By comparison, the SVM classifier could increase the learning accuracy by 7.54%, while the NN model only achieved a passable accuracy of around 91%. The learning accuracy determines the results of defect identification, and therefore, the NN model was not considered further to reconstruct the profiles.

The last procedure concerned reconstructing the inspection profiles and identifying the defects, with negative traces of all-zero vectors and positive traces output after NNs. Table 6 presents the learning accuracy of the entire developed framework, by comparing the reconstructed profiles with the ground truth images. The validation accuracy remarkably reached 99.44%, indicating the effectiveness of our learning procedures. Although the WSN+SVM procedure had a 5.26% classification error, each misclassified trace affected less than 10 pixels, and thereby, the effect on the whole framework was less than 5.26%×10/1250=0.042%. Although the SVM+NN procedure had a 1.19% error, the positive traces only accounted for around 38% of all traces. The effect on the whole framework was around 1.19%×38%=0.452%. Therefore, the ultimate learning accuracy was higher than the partial procedures.

**Figure 6:**
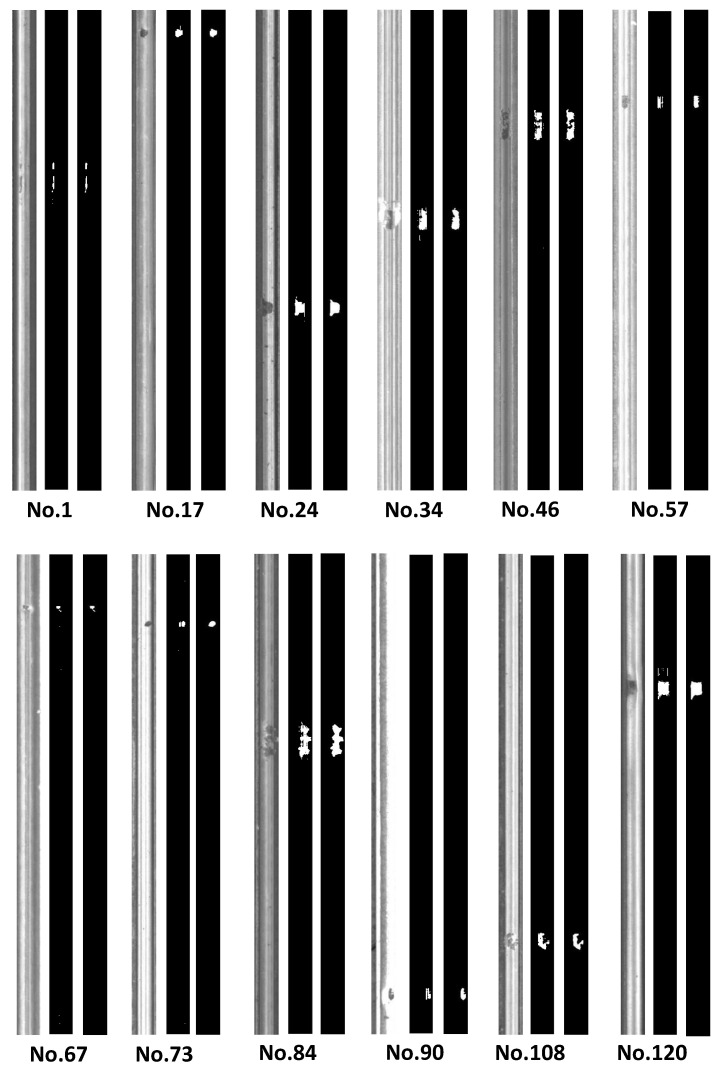
Example rails in the validation set, with labels Nos. 1, 17, 24, 34, 46, 57, 67, 73, 84, 90, 108, and 120 in the Type-II RSDD dataset. The sequence of each rail images is the original image, the learning result, and the ground truth.

Figure 6 shows the learning results of 12 example rails (the labels are provided in the dataset) in the validation set. The original images had different brightness, contrast, and defect sizes. Despite these factors, the reconstructed profiles corresponded well with the ground truth images, as the defect locations and shapes were visibly similar. For example, the identified locations of defects on the No. 1 rail were (21, 406) and (20.5, 458), while the actual locations were (21, 406) and (20.5, 454), with errors less than four pixels. The defect sizes were convenient to determine by summing the white pixels. The maximum error occurred in the No. 57 rail, as the identified defect size was 293 pixels and the error was 9.6%. The minimum error occurred in the No. 46 rail, as the identified defect size was 972 pixels and the error was 0.1%. Therefore, the developed approach performed promisingly in identifying the sizes and locations of the railhead defects.

### 3.3. Comparison with the Literature

The RSDD dataset has been extensively applied in the literature [15,17,36,38,42,43], and thereby, our defect detection approach can be properly compared with previous ones to evaluate the performance. The same evaluation criteria, namely the precision (Pre), recall (Rec), and F-measure (F), were adopted at both the pixel and defect levels. Detailed definitions were given by [17].

The pixel-level indexes, which evaluate the pixel accuracy, can be calculated with the following equations:

(17) $$\begin{array}{cc} Pre & =TP/(TP+FP)\\ Rec & =TP/(TP+FN)\\ F & =2\times Pre\times Rec/(Pre+Rec) \end{array}$$

where *TP* represents the number of correctly identified defect pixels, *FP* is the number of the non-defect pixels misidentified as defect pixels, and *FN* is the number of unrevealed defect pixels.

The defect-level indexes, which evaluate the number of correctly detected defects, can be calculated with the following equations:

(18) $$\begin{array}{cc} Pre^{\prime } & =nTP/P\\ Rec^{\prime } & =nTP^{\prime }/N\\ F^{\prime } & =2\times Pre^{\prime }\times Rec^{\prime }/\left(Pre^{\prime }+Rec^{\prime }\right) \end{array}$$

where *nTP* represents the number of correctly detected defects that have over 50% areas overlapping the labeled defects, *nTP’* represents the number of correctly detected defects that overlap more than 50% of the labeled defects, *P* is the number of detected defects, and *N* is the number of labeled defects.

Table 7 and Table 8 list the previous results in the literature and the learning results in this paper. Our learning results were calculated on the validation sets, with 24 labeled defects from the Type-I dataset and 32 labeled defects from the Type-II dataset. At the pixel level of the Type-I dataset, the developed learning approach outperformed the previous approaches, improving the precision, recall, and F-measure criteria to over 90%. The best previous performance was achieved by Gan et al. in 2017 [36] with the criteria Pre = 87.54%, Rec = 85.63%, and F = 85.12%, while the developed approach achieved further promising criteria of Pre = 93.21%, Rec = 90.80%, and F = 91.99%. The precision rate was higher than the recall rate, as the SVM classifier discarded the negative traces and thereby reduced the false positive possibility. The Type-II dataset also achieved promising results, as the developed learning approach outperformed the previous approaches, improving the precision, recall, and F-measure criteria to around 90%. The highest criteria rates obtained by the previous methods were Pre = 84.12%, Rec = 87.24%, and F = 82.11%, while our criteria rates reached Pre = 89.69%, Rec = 90.48%, and F = 90.08%. The precision rate became lower since the SVM procedure contained a 5.26% classification error, basically affected by the image quality. Nonetheless, the developed learning framework presented advanced detection performance compared to previous approaches.

The results differed a little at the defect level. The recall rates of both datasets were 100%, which means all labeled defects were detected. This was a significant improvement as previous research overlooked some challenged defects. However, the precision rates were both around 75%, which also affected the F-measure criteria, and these values were smaller than the previous best performance. Since unavoidable learning biases generated tiny speckle patterns smaller than 20 pixels (nearly invisible in Figure 5 and Figure 6), they were misidentified as independent defects affecting the precision criteria. Nonetheless, these speckles were insignificant and had little effect on identifying sizes and locations. The learning results were promising, but left room for improvement for eliminating the speckles.

## 4. Discussion and Conclusions

This paper developed a machine learning framework based on WSNs and NNs for identifying both the locations and sizes of railhead defects. These defects included squats at different levels and other related damages produced by rolling contact fatigue. Other important defects, like rail corrugation and railhead wear, were not concerned. Crossings and turnouts were also excluded in this paper. Three significant research developments were achieved: outputting size information, promising results on non-intensive datasets, and improved accuracy of railhead inspection. The defect sizes were specified on the track surface (lengths along the track direction and widths) except the depths and other parameters. Three inseparable components in the learning framework contributed to the development. Firstly, WSNs were functionally equivalent to deep CNNs for feature extraction, and they constituted a predefined model without data requirement. This ensured a high-quality characteristic output without training parameters. We also want to mention that WSNs were not compared with CNNs here since the adopted non-intensive datasets were not suitable for CNNs. Secondly, the SVM classifier was designed to sieve positive traces. This component was significant as negative traces accounted for over 60%, but provided no defect information, which would generate the illusion to the learning model that almost all areas were negative. Thirdly, NNs with specific activation functions were designed to restore the ground truth images. Different activation functions determined the nonlinear relationship between variables, thereby significant for improving learning accuracy.

The developed approach was evaluated by the publicly available RSDD datasets, with 67 images in the Type-I dataset and 128 images in the Type-II dataset. By comparing WSN+SVM with an independent SVM, WSNs could properly extract signal characteristics and improve classification accuracy up to 97.20% and 94.74% for the two datasets, respectively. The classification errors affected less than 0.05% on the final accuracy as each misclassified trace only affected fewer than 10 pixels. By comparing SVM+NN with NN, the SVM classifier could improve the learning accuracy by over 6%, although the NN models also achieved passable results. The ultimate learning accuracy reached 99.80% and 99.44% for the two datasets, respectively, higher than the partial results. The learning results of the developed model were extensively compared with the methods in the literature, utilizing three criteria, namely precision, recall, and F-measure. At the pixel level, the developed approach remarkably outperformed previous models, improving these criteria to around 90%. At the defect level, the recall rates reached 100%, indicating all labeled defects were identified. The precision rates were around 75%, affected by the insignificant misidentified speckles (smaller than 20 pixels). Nonetheless, the developed learning approach was effective in identifying railhead defects.

Further research will focus on three aspects. Firstly, automatic approaches should be complemented to eliminate misidentified speckles. Secondly, the developed learning model should be evaluated by extensive datasets with different image qualities. Thirdly, this machine learning approach will be developed for real-time railhead inspection. Fourthly, the possibility of camera inspection assisted by machine learning for the wear and corrugation defects will be investigated.

## Figures and Tables

**Table 1:** Accuracy and false rates of classifying vertical traces with or without the WSN (Type-I dataset).

Models	Training Accuracy	Accuracy	Validation FP	FN
WSN+SVM	99.95%	97.20%	0.93%	1.87%
SVM	99.95%	72.43%	0.05%	27.52%

**Table 2:** Accuracy and false rates of restoring ground truth traces with or without the SVM (Type-I dataset).

Models	Training Accuracy	Accuracy	Validation FP	FN
SVM+NN	99.84%	99.47%	0.19%	0.34%
NN	92.43%	92.16%	1.75%	6.09%

**Table 3:** Accuracy and false rates of reconstructing profiles by the developed framework (Type-I dataset).

Models	Training Accuracy	Accuracy	Validation FP	FN
Developed model	99.96%	99.80%	0.08%	0.12%

**Table 4:** Accuracy and false rates of classifying vertical traces with or without WSN (Type-II dataset).

Models	Training Accuracy	Accuracy	Validation FP	FN
WSN+SVM	99.57%	94.74%	1.99%	3.27%
SVM	99.52%	68.96%	0.50%	30.54%

**Table 5:** Accuracy and false rates of restoring ground truth traces with or without SVM (Type-II dataset).

Models	Training Accuracy	Accuracy	Validation FP	FN
SVM+NN	99.77%	98.81%	0.64%	0.55%
NN	91.40%	91.27%	1.71%	7.02%

**Table 6:** Accuracy and false rates of reconstructing profiles by the developed framework (Type-II dataset).

Models	Training Accuracy	Accuracy	Validation FP	FN
Developed model	99.85%	99.44%	0.27%	0.29%

**Table 7:** Comparison with the literature (Type-I dataset). Pre, precision; Rec, recall.

Approach	Pre	Rec	F	Pre’	Rec’	F’
Li and Ren 2012 [15]	-	-	-	76.26%	70.80%	73.43%
Li and Ren 2012 [42]	78.14%	78.89%	75.70%	47.87%	85.40%	61.35%
He et al., 2016 [43]	-	-	-	41.19%	72.94%	61.35%
Gan et al., 2017 [36]	87.54%	85.63%	85.12%	93.02%	85.40%	89.05%
Yu et al., 2018 [17]	86.54%	77.68%	80.02%	84.06%	77.37%	80.58%
Nieniewski 2020 [38]	80.91%	83.14%	78.77%	64.48%	86.67%	73.95%
	81.54%	82.62%	78.80%	68.52%	80.00%	73.82%
	80.87%	83.04%	78.67%	65.73%	86.67%	73.95%
	81.56%	82.45%	78.68%	69.87%	78.52%	73.94%
Developed approach	93.21%	90.80%	91.99%	75.00%	100%	85.71%

**Table 8:** Comparison with the literature (Type-II dataset).

Approach	Pre	Rec	F	Pre’	Rec’	F’
Li and Ren 2012 [15]	-	-	-	88.89%	71.82%	79.45%
Li and Ren 2012 [42]	73.88%	83.05%	76.05%	58.68%	91.71%	71.56%
He et al., 2016 [43]	-	-	-	49.73%	46.41%	48.01%
Gan et al., 2017 [36]	83.88%	83.58%	82.11%	91.91%	83.98%	87.76%
Yu et al., 2018 [17]	84.12%	73.25%	76.45%	85.83%	83.98%	84.89%
Nieniewski 2020 [38]	73.18%	87.24%	77.08%	72.04%	90.68%	80.30%
	72.41%	87.07%	76.61%	72.78%	90.68%	80.75%
	73.22%	87.20%	77.06%	78.61%	90.68%	84.22%
	72.57%	87.07%	76.61%	82.25%	89.44%	85.70%
Developed approach	89.69%	90.48%	90.08%	75.61%	100%	86.11%

## Data Availability

Data available in a publicly accessible repository that does not issue DOIs Publicly available datasets were analyzed in this study. This data can be found here: http://icn.bjtu.edu.cn/visint/resources/RSDDs.aspx (accessed on 5 April 2021).
